# Vegan Triple-Ironman (Raw Vegetables/Fruits)

**DOI:** 10.1155/2014/317246

**Published:** 2014-01-12

**Authors:** Roman Leischik, Norman Spelsberg

**Affiliations:** Department of Prevention and Sports Medicine, University of Witten/Herdecke, Germany

## Abstract

Endurance sport requires a healthy and balanced diet. In this case report we present the findings of an ultra-triathlete (three times Ironman, means 11.4 km swim, 540 km bike, 125 km run in 41:18 h as a whole) living on a raw vegan diet and having finished the competitions under these nutritional conditions. To this end, the vegan ultra triathlete and a control group of 10 Ironman triathletes of similar age living on a mixed diet were investigated, using echocardiography and spiroergometry. In addition, blood samples were taken from the vegan athlete both in the sporting season and in the off-season. The vegan athlete showed no signs of dietary deficiencies or impaired health. In comparison with the control group, the vegan athlete showed a higher oxygen intake at the respiratory compensation point. This case demonstrates that even top-class sporting performance, like that of a three-time Ironman, is possible on a vegan diet. Whether a vegan diet offers advantages or disadvantages for the performance of endurance athletes remains an open question.

## 1. Case Report

A 48-year-old male finished Triple-Ironman distance in 41 hours and 18 minutes (11.4 km swimming, 540 km cycling, and 126 km running). At the time of the examinations, he had been practising his current diet of raw vegan diet for 6 years. Prior to this, the vegan athlete had been living as a vegan for 3 years and as a vegetarian for the previous 13 years.

All last competitions were performed only based on a raw diet. At the time of both examinations, the vegan athlete was 48 years of age and 1.80 metres in height. In the sporting season he was 79.7 kg in weight, with a body fat index of 12.9%; in the off-season he weighed 80.3 kg with a body fat index of 16.3%. Clinical examination showed a regular heart rhythm at 60 beats/min. Blood pressure was 115/70. The heart sounds were normal.

Prior to the spiroergometry, echocardiography was performed based on ASA criteria (1).

For comparison purposes, we refer to the values for 10 Ironman triathletes of similar age living on a mixed diet.

The results of the spiroergometry are shown in Table 1, those of echocardiography are in Table 2, and blood analysis findings are presented in Table 3. The athletes of the control group were aged 47.4 ± 5.2, weighed 76.2 ± 8.9 kg (with 13.4 ± 2.0% body fat), and were 1.816 metres ±6.6 cm in height. In the active phase the vegan athlete was training on average 18 hours per week, consisting of 2 hours of swimming, 11 hours of cycling, and 5 hours running. This involved covering distances of 5 km (swimming), 330 km (cycling) and 50 km (running). The athletes of the control group were training for a total of 15.9 ± 2.1 hours weekly, involving 2.5 ± 0.7 hours and 5.6 ± 1.5 km swimming, 8.6 ± 1.5 hours and 215.5 ± 53.0 km cycling, and 4.9 ± 0.7 hours and 55.2 ± 6.5 km running.

In terms of performance diagnostics, the vegan athlete showed comparable VO_2_max, VO_2_ at VAT, and %VO_2_max at VAT values as compared with the control group (statements regarding significant differences are not possible). VO_2_ and %VO_2_max at RCP were somewhat higher for the vegan athlete. The maximum ergometric performance is higher for the vegan athlete in absolute terms but not relative to the body weight. At RCP the ergometric performance of the vegan athlete is somewhat higher than that of the control group. The vegan athlete had lower pulse rates at rest, at VAT, at RCP, and at the endurance limit. In comparison with the off-season, in the active season the vegan athlete had a higher maximum ergometric performance, VO_2_, and %VO_2_max, as well as cardiac frequency at RCP.

In morphological terms, the vegan athlete showed a greater left ventricular end diastolic diameter, with consecutively higher end diastolic volume and stroke volume. The systolic and diastolic functions seem to be similar for the vegan athlete and the control group.

Besides a mild thrombopenia, slightly higher CK-NAC levels in the active season and a slight drop of free testosterone (in both examinations) all remaining values were within normal ranges.

## 2. Discussion

It is well known that a vegetarian or vegan diet, when sensibly managed, can make a contribution to the prevention and therapy of illnesses in all phases of life [1]. More and more people are thus adopting a vegetarian (2) or vegan (2) lifestyle. It is equally understandable that such people may want to take part in endurance sports. It follows that the effects of vegan diet on sporting performance are a matter of general interest.

This case report is set out to investigate an ultra endurance triathlete, who had been living on a vegetarian diet since 22 years and a purely vegan diet for 9 years, with reference to his ability to perform, cardiac status, and any symptoms of deficiency. It was also a matter of interest for the study to ascertain whether his performance parameters were different from those of mixed diet triathletes.

Even competitive athletes can adopt a diet of this kind with a view to the health benefits, without any risk of suffering from dietary deficiencies [2–4].

With its disciplines of swimming, cycling, and running, the triathlon is a very popular type of endurance sport. As well as competitions involving classic sprint distances (0.5, 20 and 5 km), short distances (1–1.5, 40, and 10 km), and medium distances (ca. 2, 80–90, and 21.2 km), it also offers competitions involving ultra distances: Ironman (3, 8, 180, and 42.2 km) and multiple (2× until 10×) Ironman.

As is the case with other types of endurance sport, triathletes undergo various physical adaptations in the course of training. These include an increased cardiac output under stress and increased arteriovenous oxygen extraction with consecutive rises in the maximum oxygen intake (VO_2_max) [5]. In echocardiographic terms, triathletes show physical changes similar to those of cyclists, resembling a mixed state of static/dynamic stress and involving a significant increase in the left ventricular muscle mass and internal left ventricular diameter [6]. In terms of spiroergometry, successful triathletes not only show an increased oxygen intake, but they also demonstrate a capacity for continuous high performance over long periods, as a result of effective internal economy, high fractional extraction of VO_2_max at the given stress level (%VO_2_max), and a high anaerobic threshold [7]. Good indicators of the limit of endurance are the ventilatory anaerobic threshold (VAT) and the respiratory compensation point (RCP) [7, 8].

It is not known whether or to what extent triathletes living on a vegan or vegetarian diet differ from triathletes living on a mixed diet. It is known that nonsporting vegans show better metabolic parameters than mixed diet athletes (9). To the best of our knowledge, no data are to be found in the extant literature on the spiroergometric, echocardiographic, or haematological parameters of ultra endurance athletes living on a vegan diet. As a result, this is a ground-breaking case report involving the first ever presentation of the characteristic parameters of a vegan ultra endurance athlete.

In view of the equivalence in terms of age and other similar anthropometric features, the data for the vegan athlete are well comparable with those of the control group. On the other hand, we are dealing here with an individual case, so the meaningfulness of the results, in the nature of the case, remains limited. Both the vegan athlete and the control group trained for a comparable number of hours. The vegan athlete's cycling training, on the other hand, was more intense and more prolonged. Comparison of the competition results would be a way of checking this, but in view of the fact that the athletes' preferred competition distances vary from one another, this is not possible. Here, however, we should draw attention to the considerably different age of the athletes. Millet et al. [8] report on athletes of 24.8 ± 2.6 years of age. In comparison with the off-season, in the active season the RCP and the maximum ergometric performance rose moderately, whereas the VAT and VO_2_max remained at a constant level. This study demonstrated a significant rise in the RCP and maximum ergometric performance (with VAT and VO_2_max remaining constant), based on comparison between the pre-competitive and competitive seasons.

The echocardiographic parameters of the vegan athlete are different from those of the control group. Essentially the vegan athlete has a greater LVEDD value, with higher EDV and SV, which however do not exceed standard and normal values [9]. Overall the echocardiographic values of the sporting season and the off-season do not differ in any significant degree.

Laboratory parameters showed no pathological values. A slight thrombopenia in the sporting season can be explained by the reduced thrombocyte activation and adhesion caused by training. In itself this is not necessarily a pathological manifestation. In conjunction with a threshold value for vitamin B12 and standard iron, ferritin, and folic acid status, the standard haemoglobin, haematocrit, MCH, MCV, and MCHC readings exclude the possibility of manifest or latent anaemia. Raised CK-NAC levels are not unusual with active athletes. Mougious et al. have therefore written about the necessity of separate CK reference values for active sportspersons. Reduced testosterone values in the blood have also been described. An influence on the bone density or the muscle mass, however, cannot be demonstrated. None of the usual laboratory parameters gives grounds for suspecting the presence of any detriment to health caused by sport or by diet. Quite on the contrary, the vegan athlete even shows ideal cholesterol values.

## 3. Critical Remarks

The case report of a vegan triathlete can only provide indications that a vegan diet is at least not detrimental to health, even in connection with a long-distance triathlon, and that a vegan diet results in similar performance as compared with a mixed diet. These conclusions cannot be extrapolated to short distances and Olympic distances, where an explosive performance—above all, a performance in the lactic acid zone—is frequently necessary. Taking place for the most part in the aerobic range, long-distance races may work more in favour of vegan diet than races that approach the anaerobic threshold. In view of the shortage of suitable and willing test subjects, it will not be easy to arrange a group comparison of vegan and traditional triathletes for study purposes. A case study like the present one can supply indications, but the meaningfulness of its findings remains subject to limit.

This case report seems to suggest that an ultra triathlete living on a vegan diet has a similar profile, physiologically and in performance terms, to that of triathletes of similar age living on a conventional diet. The cardiac adaptations are likewise similar in both cases. The higher laboratory parameters do not permit conclusions with regard to dietary deficiency or any detriment to health. A well-planned vegan diet can apparently also be adopted by ultra endurance athletes without any risk to health necessarily being incurred. To what extent a vegan diet offers benefits or disadvantages for the performance capacity/health of a triathlete would be a matter for investigation in a future study, though in view of the highly individual training regimes of triathletes, this would be difficult to arrange.

## Figures and Tables

**Table 1:** Spiroergometric parameters of the vegan athlete and the control group (values given as a mean value ± standard deviation).

		Vegan Off-S.	Vegan season	Control group (*n* = 10)
VO_2_ max	L/min	4.8	4.7	4.3 ± 0.6
	mL∗min^−1^∗kg^−1^	60.0	60.0	56.9 ± 7.5

VO_2_ VAT	L/min	3.4	3.3	3.3 ± 0.6
	mL∗min^−1^∗kg^−1^	43	42	43.7 ± 7.4
	% VO_2_ max	71	70	76.1 ± 9.3

VO_2_ RCP	L/min	3.9	4.4	3.6 ± 0.6
	mL∗min^−1^∗kg^−1^	48	55	48.2 ± 8.3
	% VO_2_ max	80.6	92	84.4 ± 6.9

HR	At rest	55	49	58.6 ± 2.9
	At VAT	127	123	147.7 ± 11.4
	At RCP	141	147	157.0 ± 13.1
	Maximal load	163	159	176.7 ± 11.0

W	At VAT	280	280	262 ± 42.9
	At RCP	340	370	286 ± 56.2
	Maximal load	370	400	337 ± 53.8
	Maximal load/kg body weight	4.61	5.02	4.46 ± 0.73

HF: heart rate/min, Off-S: off-season, season: active season, vegan: vegan athlete, VO_2_ max: maximum oxygen intake, VO_2_ VAT: oxygen intake at ventilatory anaerobic threshold, VO_2_ RCP: oxygen intake at respiratory compensation point, and W: ergometric performance.

**Table 2:** Echocardiographic parameters.

		Vegan athlete, off-season	Vegan athlete, active season	Control group (*n* = 10)
Aortic root	cm	3.7	3.4	3.1 ± 0.4
Left atrium	cm	3.0	3.0	2.6 ± 0.3
Left atrium	ml	36	30	29.6 ± 8.6
LVEDD	cm	5.0	5.4	4.64 ± 0.4
IVSD	cm	1.2	1.1	1.2 ± 0.1
PWTD	cm	1.2	1.0	1.2 ± 0.1
LVMM	g	211.3	206.3	203.0 ± 35.3
LVMM	g/m²	105.68	103.67	103.1 ± 14.5
EDV	mL	119	140	100.2 ± 16.7
ESV	mL	48	58	39 ± 6.1
SV	mL	72	82	61.2 ± 11.2
EF	%	60	59	61.0 ± 2.1
SF	%	32	31	35.5 ± 9.4
MV E/A ratio		1.45	1.56	1.39 ± 0.20

EDV: end diastolic volume, EF: ejection fraction, ESV: end systolic volume, IVSD: intraventricular septum in diastole, LVEDD: left ventricular end diastolic diameter, LVMM: left ventricular muscle mass, MV E/A ratio: mitral valve E/A ratio, PWTD: posterior wall thickness in diastole, SF: shortening fraction, and SV: stroke volume.

**Table 3:** Results of blood investigation of the vegan ultra triathlete.

Analysis	Off-season	Active season
Leukocytes (1000 s/uL)	4.6	4.7
Erythrocytes (Mill·uL)	5.1	4.9
Haematocrit (%)	43	42
Haemoglobin (g/dL)	14.9	14.1
MCV (fl)	83	87
MCH (pg)	29.0	29.1
MCHC (g/dL)	35.1	33.3
Thrombocytes (1000 s/uL)	171	138
Alkaline phosphatase (U/L)	68	70
Glutamyl transpeptidase	8	15
Glutamate oxaloacetate transaminase (U/L)	36	40
Glutamate pyruvate transaminase (U/L)	32	42
Lactate dehydrogenase (U/L)	199	238
Amylase (U/L)	63	61
Lipase (U/L)	35	36
CK-NAC (U/L)	155	209
Protein (g/dL)	6.6	6.7
Cholesterol (mg/dL)	134	113
HDL (mg/dL)	47	51
LDL (mg/dL)	74	50
Triacylglyceride (mg/dL)	148	84
TSH basal (mU/L)	1.23	0.73
Iron (*µ*g/dL)	95.0	82.0
Free testosterone (pg/mL)	5.5	5.7
Vitamin B12 (pg/mL)	329	347
Ferritin (ng/mL)	83.7	82.6
Folic acid (ng/mL)	16.8	14.3
